# Mediating Effect of Repeated Tuberculosis Exposure on the Risk of Transmission to Household Contacts of Multidrug-Resistant Tuberculosis Patients

**DOI:** 10.4269/ajtmh.17-0325

**Published:** 2017-12-04

**Authors:** Peng Lu, Xiaoyan Ding, Qiao Liu, Wei Lu, Leonardo Martinez, Jiansheng Sun, Feng Lu, Chongqiao Zhong, Hui Jiang, Changdong Miao, Limei Zhu, Haitao Yang

**Affiliations:** 1 Department of Chronic Communicable Disease, Center for Disease Control and Prevention of Jiangsu Province, Nanjing, Jiangsu Province, People’s Republic of China;; 2 Department of Epidemiology and Biostatistics, University of Georgia School of Public Health, Athens, Georgia;; 3 School of Medicine, Division of Infectious Diseases and Geographic Medicine, Stanford University, Stanford, California;; 4 Department of Chronic Communicable Disease, Center for Disease Control and Prevention of Xuzhou City, Jiangsu Province, China;; 5 Department of Chronic Communicable Disease, Center for Disease Control and Prevention of Nantong City, Jiangsu Province, China;; 6 Department of Chronic Communicable Disease, Center for Disease Control and Prevention of Lianyungang City, Jiangsu Province, China;; 7 Department of Chronic Communicable Disease, Center for Disease Control and Prevention of Zhenjiang City, Jiangsu Province, China;; 8 Department of Chronic Communicable Disease, Center for Disease Control and Prevention of Taizhou City, Jiangsu Province, China;; 9 Jiangsu Institute of Parasitic Diseases, Jiangsu Province, China

## Abstract

Primary *Mycobacterium tuberculosis* transmission is an important driver of the global epidemic of resistance to tuberculosis drugs. A few studies have compared tuberculosis infection in contacts of index cases with different drug-resistant profiles, suggesting that contacts of multidrug-resistant (MDR) tuberculosis cases are at higher risk. Repeated tuberculosis exposure in contacts of MDR tuberculosis patients through recurrent tuberculosis may modify this relationship. We compared tuberculosis infection in household contacts of MDR and drug-susceptible (DS) tuberculosis patients from six cities in southeastern China and investigated whether repeated tuberculosis exposure was a mediating factor. Tuberculosis infection was defined as a tuberculin skin test induration ≥ 10 mm. In all, 111 (28.0%) of 397 household contacts of MDR tuberculosis patients and 165 (24.7%) of 667 contacts of DS tuberculosis index cases were infected with tuberculosis. In a multivariate model not including the previous tuberculosis exposure, contacts of MDR tuberculosis patients had a higher likelihood of tuberculosis infection (adjusted odds ratio [AOR] = 1.37; 95% confidence interval [CI] = 1.01–1.84; *P* = 0.041). In a separate multivariate model adjusted for the previous tuberculosis exposure, the odds ratio of tuberculosis infection flipped and contacts of MDR cases were now at lower risk for tuberculosis infection (AOR = 0.55; 95% CI = 0.38–0.81; *P* = 0.003). These findings suggest prior tuberculosis exposure in contacts strongly mediates the relationship between tuberculosis infection and the index drug resistance profile. Prior studies showing lower risk of developing tuberculosis among contacts of MDR tuberculosis patients may be partially explained by a lower rate of tuberculosis infection at baseline.

## INTRODUCTION

Drug-resistant tuberculosis has the potential to substantially impede current and future efforts to control the global tuberculosis epidemic. Resistance to tuberculosis drugs leads to reduced treatment effectiveness and overall elevated costs in tuberculosis treatment and, due to this, multidrug-resistant (MDR) tuberculosis patients are more likely to have catastrophic costs, adverse health outcomes, and mortality compared with drug-susceptible (DS) tuberculosis patients.^1^ China has the most MDR patients globally and primary transmission is largely responsible for increasing rates of drug resistance in recent years.^2,3^ A further understanding of the transmission dynamics of drug-resistant tuberculosis patients to susceptible contacts in their social network is necessary to implement effective policy that can assist in blunting the spread of the epidemic.

The comparative transmission potential of MDR and DS tuberculosis patients is controversial.^4^ Several studies argue that MDR tuberculosis patients are less likely to transmit to their social network compared with DS tuberculosis patients because of potential fitness costs through genetic mutations linked to resistance.^5–9^ Tuberculosis infection in household contacts is used to compare the fitness of mycobacteria tuberculosis.^10–12^ Few studies have compared rates of tuberculosis infection in household contacts of MDR and DS tuberculosis patients and these suggest contacts of MDR patients have higher levels of tuberculosis infection.^9–13^ Two of these studies^10,11^ found higher levels of tuberculosis infection among contacts of MDR tuberculosis patients but this difference did not reach statistical significance. Three other studies^9,12,13^ found a statistical higher rate of tuberculosis infection among contacts of MDR patients.

A possible reasoning for these results may be the disproportionate number of tuberculosis disease episodes among index cases. MDR patients are much more likely to have several disease events, sometimes as much as three times more than DS patients,^8^ and therefore they may be infectious for longer periods of time compared with DS patients. In these previous studies, rates of tuberculosis infection among these two groups were not adjusted for multiple tuberculosis episodes among MDR tuberculosis patients. We conducted a large household contact investigation and compared the prevalence of tuberculosis infection in contacts of MDR versus DS tuberculosis index cases in six cities in China, a country with the highest number of drug-resistant cases globally.^2^ We hypothesized that differential rates of previous tuberculosis episodes among MDR and DS tuberculosis index patients would mediate the association between tuberculosis infection in household contacts and the index’s drug resistance profile.

## MATERIALS AND METHODS

### Study design and population.

Between December 2011 and December 2014, we recruited tuberculosis index cases and their household contacts in six cities throughout Jiangsu province, China. Index cases were defined as the first presenting tuberculosis case in the household. All tuberculosis patients were diagnosed in six tuberculosis-designated hospitals through clinical examinations, radiographical imaging, microscopic sputum smear, sputum culture, and drug sensitivity testing. Tuberculosis patients were classified as either MDR or DS using conventional culture-based drug susceptibility testing. MDR tuberculosis patients were defined as those who were resistant to at least isoniazid and rifampicin in vitro. DS tuberculosis patients were matched to each confirmed MDR tuberculosis index patient by the same region and the closest diagnostic time. Tuberculosis patients with both sputum smear and culture negative laboratory results were excluded. Enrolled index cases were subsequently interviewed and demographic and clinical characteristics were collected, including age, sex, smear status, and smoking status. Smoking status was self-reported by participants.

After index case interviews, all households were visited by trained field workers and nurses. Household contacts were defined as any individual spending at least seven consecutive days in the same household as the index case ≤ 3 months before diagnosis and ≤ 14 days after initiating therapy. Pregnant women, nursing mothers, or those with active tuberculosis were excluded. All household contacts were recruited regardless of age and, of those that consented, were interviewed through structured sociodemographic and clinical questionnaires. Information was collected on household contacts, including age, sex, Bacillus Calmette-Guérin (BCG) vaccination history, past tuberculosis, and smoking status. Participants were inspected for Deltoid scars compatible with BCG vaccination. Environmental characteristics were recorded, including family size, the number of bedrooms in the household, region in the province, presence of air conditioning, or the presence of an independent kitchen. Previous tuberculosis exposure of household contacts was obtained through detailed questioning of both index cases and household contacts and was defined as previous exposure to other tuberculosis cases, either to the present index case (through tuberculosis recurrence) or from exposure to another individual with tuberculosis.

A tuberculin skin test was performed by trained nurses in accordance with standard national guidelines, using intradermal injection of 0.1 mL of 5 tuberculin units purified protein derivative (Guangzhou Longcheng Pharmaceutical Co., Ltd, Guangzhou City, China).^14^ Tuberculin skin test results were read 48–72 hours after administration, and the diameter of induration was measured transversely on the forearm of each contact using the Mantoux method.^14^ A positive tuberculin skin test was defined as an induration reaction ≥ 10 mm.^15^

### Data analytical plan.

Two investigators double-entered the data using EpiData 3.1 software (Odense, Denmark) to confirm consistency and accuracy. When conflicting entries were identified, a third reviewer would examine the original questionnaires. For exploratory data analysis, we summarized continuous variables as medians with interquartile ranges (IQRs) and categorical variables using standard 2 × 2 contingency tables. We used the Pearson χ^2^ and Fisher exact tests as appropriate to derive *P* values for categorical variables. We then stratified tuberculosis infection by index case, household contact, and environmental characteristics. A binary univariate logistic regression model was used to calculate odds ratios (ORs) for tuberculosis infection and all included characteristics.

We then built a multivariable regression model. *A priori*, we included several variables into the multivariate model regardless of *P* value because of our research question and established associations reported in the literature. These variables included contact age, sex, and the drug resistance profile (MDR versus DS) of the index case. We then began adding variables one at a time that were suggestively related to tuberculosis infection in univariate analysis (*P* < 0.20). Because we hypothesized that previous tuberculosis exposure may mediate the rate of tuberculosis infection among household contacts with differing resistance profiles, we aimed to quantify the direct effect of index MDR status on tuberculosis infection in contacts. To investigate whether previous tuberculosis exposure mediated the relationship between tuberculosis infection in household contacts of MDR and DS tuberculosis index cases, we performed and compared two multivariable models: 1) in the first regression model, we included all covariates except prior tuberculosis exposure of the contact and 2) in a separate multivariate model, we added the mediating variable (prior tuberculosis exposure) in addition to all other covariates. This second model quantifies the direct effect of index MDR status on tuberculosis infection of contacts that is not mediated by prior tuberculosis exposure. We then compared adjusted ORs for tuberculosis infection and the drug-resistant profile of the index case in each model. All statistical analysis was conducted using the SPSS software (version 23.0, IBM Corporation, Armonk, NY).

### Ethical considerations.

This study was reviewed and approved by the Ethics Committee of Jiangsu Province Center for Disease Control and Prevention. The study was conducted in accordance with approved guidelines, and written informed consent was obtained from all eligible tuberculosis patients and all enrolled household contacts.

## RESULTS

### Study population.

**Figure 1.**
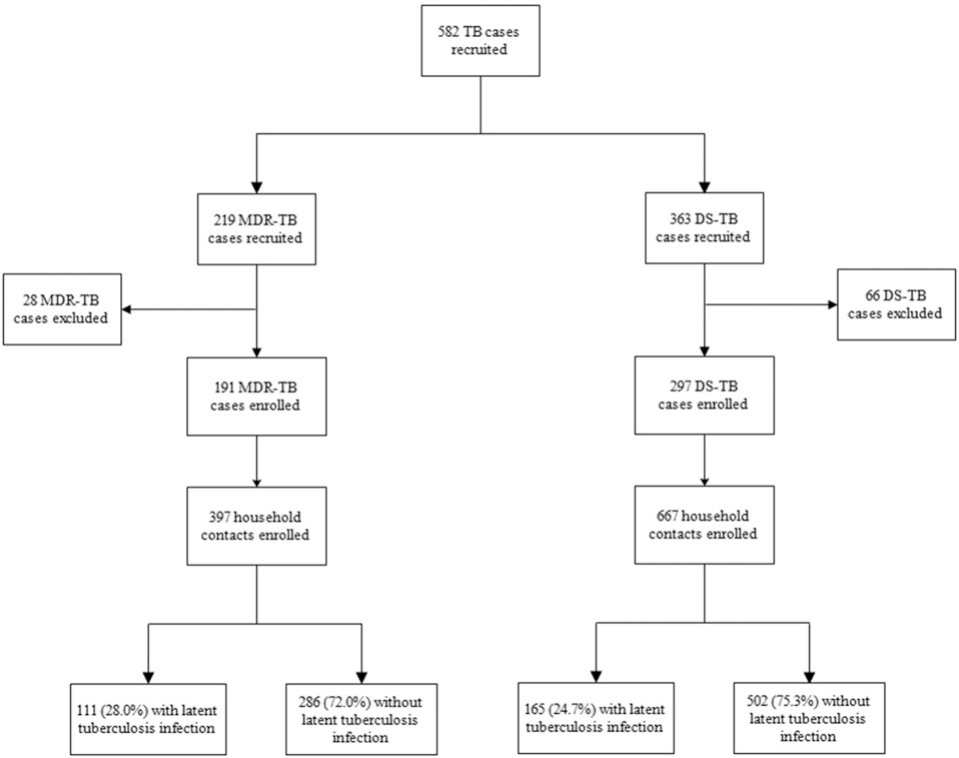
Flow diagram of recruitment and outcomes of tuberculosis infection.

A total of 219 and 363 MDR and DS tuberculosis patients were recruited in our study. Among MDR tuberculosis patients, 28 (12.8%) were excluded because they did not have household contacts, were lost to follow-up, or withdrew consent. Among DS tuberculosis patients, 66 (18.2%) were excluded due to ineligibility or because their household contacts were not administered using a tuberculin skin test. After these exclusions, 397 household contacts of 191 MDR tuberculosis patients and 667 contacts of 297 DS tuberculosis patients were enrolled (Figure 1). There were means of 2.1 and 2.2 contacts per MDR and DS tuberculosis index case, respectively.

### Risk of tuberculosis infection among household contacts.

Household contacts of MDR and DS tuberculosis patients were statistically similar in age (*P* = 0.062) and gender (40.6% versus 42.7% males, *P* = 0.487). The median age of contacts of MDR and DS tuberculosis patients was 40 (IQR = 24–52) and 43 (IQR = 24–54) years, respectively. Contacts with differing drug susceptibility profiles were also similar regarding BCG vaccination (*P* = 0.346), smoking status (*P* = 0.553), past tuberculosis (*P* = 0.569), and having an independent kitchen (*P* = 0.781). Contacts of DS cases had more bedrooms (*P* = 0.044) and more household members (*P* = 0.001) compared with contacts of MDR tuberculosis cases. However, household contacts of MDR cases were much more likely to have previous exposure to a tuberculosis case (68.3% versus 15.3%, *P* < 0.001; Table 1).

**Table 1:** Demographic characteristics of 1,064 household contacts of tuberculosis cases stratified by the drug resistance profile of the index case

Variable	Household contacts of multidrug-resistant tuberculosis cases (*n* [%])	Household contacts of drug-susceptible tuberculosis cases (*n* [%])	All contacts (*n* [%])
*N*	397 (37.3)	667 (62.7)	1,064 (100)
Age group, year			
≤ 15	75 (18.9)	116 (17.4)	191 (18.0)
15–42	139 (35.0)	195 (29.2)	334 (31.4)
≥ 42	183 (46.1)	356 (53.4)	539 (50.7)
Sex			
Male	161 (40.6)	285 (42.7)	446 (41.9)
Female	236 (59.4)	382 (57.3)	618 (58.1)
BCG vaccinated			
Yes	278 (70.0)	485 (72.7)	763 (71.7)
No	119 (30.0)	182 (27.3)	301 (28.3)
Region of province			
South	110 (27.7)	133 (19.9)	243 (22.8)
Middle	74 (18.6)	143 (21.4)	217 (20.4)
North	213 (53.7)	391 (58.6)	604 (56.8)
Previous tuberculosis exposure			
No	126 (31.7)	565 (84.7)	691 (64.9)
Yes	271 (68.3)	102 (15.3)	373 (35.1)
Family members			
≤ 4	307 (77.3)	455 (68.2)	762 (71.6)
> 4	90 (22.7)	212 (31.8)	302 (28.4)
Independent kitchen			
No	42 (10.6)	67 (10.0)	109 (10.2)
Yes	355 (89.4)	600 (90.0)	955 (89.8)
Bedrooms			
≤ 4	321 (80.9)	503 (75.4)	824 (77.4)
> 4	76 (19.1)	163 (24.4)	239 (22.5)
Missing	0 (0)	1 (0.2)	1 (0.1)
Smoking			
Yes	75 (18.9)	136 (20.4)	211 (19.8)
No	322 (81.1)	531 (79.6)	853 (80.2)
Past tuberculosis			
Yes	6 (1.5)	7 (1.0)	13 (1.2)
No	391 (98.5)	660 (99.0)	1,051 (98.8)

BCG = Bacille Calmette-Guérin.

**Figure 2.**
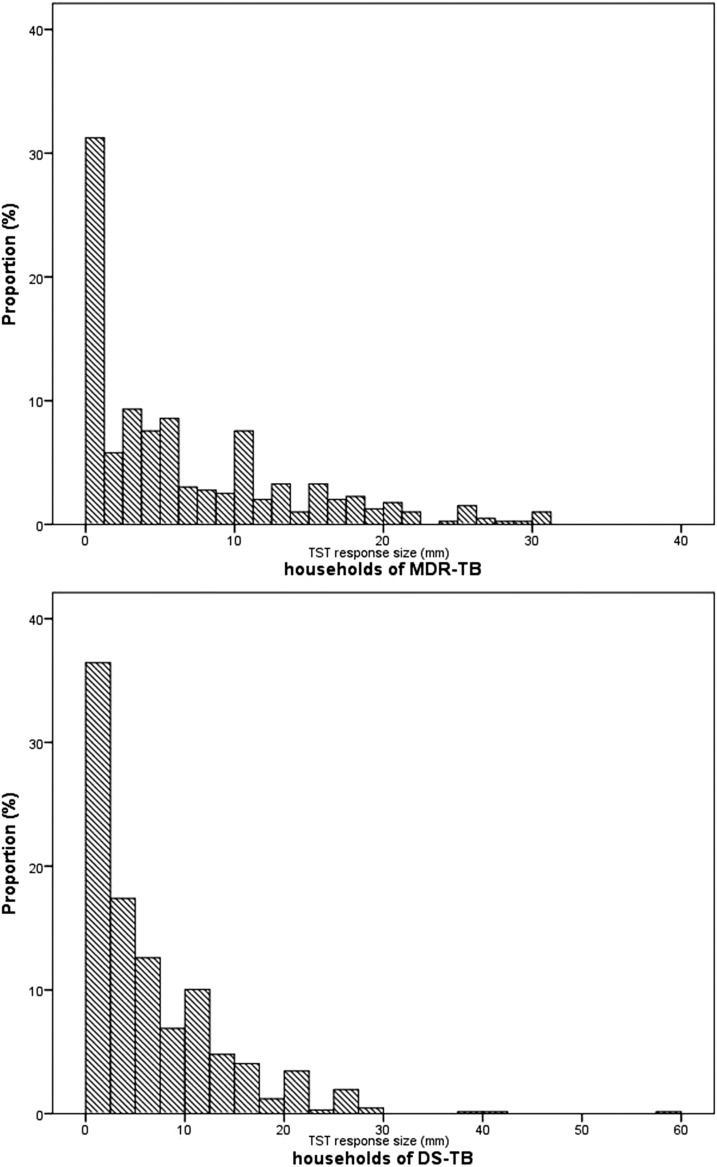
Distribution of tuberculin skin test responses among household contacts of multidrug-resistant (MDR) and drug-susceptible (DS) tuberculosis.

The frequency distribution of tuberculin skin test induration reactions in household contacts of MDR and DS tuberculosis patients is shown in Figure 2. Of 1,064 household contacts, the prevalence of tuberculosis infection was 25.9% (*N* = 276). The prevalence of tuberculosis infection among contacts of MDR and DS tuberculosis patients was 28.0% (111/397) and 24.7% (165/667), respectively.

In univariate analysis, risk factors for tuberculosis infection among household contacts included > 4 household members (OR = 1.47; 95% confidence interval [CI] = 1.10–1.98; *P* = 0.010 when compared with ≤ 4 household members), the middle region of Jiangsu province (OR = 6.91; 95% CI = 4.16–11.47; *P* < 0.001 compared with living in the southern region), northern region of Jiangsu province (OR = 3.51; 95% CI = 2.20–5.59; *P* < 0.001 compared with living in the southern region), and previous tuberculosis exposure (OR = 3.08; 95% CI = 2.32–4.09; *P* < 0.001). Participants with more than four bedrooms in the household had more tuberculosis infection but this did not reach a statistical significance (OR = 1.31; 95% CI = 0.95–1.80; *P* = 0.096 compared households with ≤ 4 bedrooms). Having an independent kitchen was a protective factor of tuberculosis (OR = 0.59; 95% CI = 0.39–0.90; *P* = 0.014) (Table 2).

**Table 2:** Risk factors for tuberculosis infection among household contacts of tuberculosis cases

Variable	No. of household contacts	No. tuberculosis infection (%)	Univariable model (*N* = 1,064)	
			cOR (95% CI)	*P* value
*N*	1,064	276 (25.9)	–	–
Age				0.967
< 15	191	50 (26.2)	Reference	–
15–42	334	88 (26.3)	1.01 (0.67–1.51)	0.966
≥ 42	539	138 (25.6)	0.97 (0.67–1.41)	0.876
Sex				
Male	446	116 (26.0)	Reference	–
Female	618	160 (25.9)	0.99 (0.75–1.31)	0.965
Drug resistance profile of index case				
Drug-susceptible	667	165 (24.7)	Reference	–
Multidrug-resistant	397	111 (28.0)	1.18 (0.89–1.56)	0.246
Region of Jiangsu Province				< 0.001
South	243	23 (9.5)	Reference	–
Middle	217	91 (41.9)	6.91 (4.16–11.47)	< 0.001
North	604	162 (26.8)	3.51 (2.20–5.59)	< 0.001
Previous tuberculosis Exposure				
No	691	125 (18.1)	Reference	–
Yes	373	151 (40.5)	3.08 (2.32–4.09)	< 0.001
BCG vaccinated				
Yes	763	206 (27.0)	Reference	–
No	301	70 (23.3)	0.82 (0.60–1.12)	0.210
No. family members				
≤ 4	762	181 (23.8)	Reference	–
> 4	302	95 (31.5)	1.47 (1.10–1.98)	0.010
Independent kitchen				
No	109	39 (35.8)	Reference	–
Yes	955	237 (24.8)	0.59 (0.39–0.90)	0.014
No. bedrooms				
≤ 4	824	204 (24.8)	Reference	–
> 4	239	72 (30.1)	1.31 (0.95–1.80)	0.096
Smoking				
Yes	211	58 (27.5)	Reference	–
No	853	218 (25.6)	0.91 (0.65–1.27)	0.567

BCG = Bacille Calmette-Guérin; cOR = crude odds ratio.

We performed two multivariate analyses. Our first multivariate analysis aimed to quantify the indirect effect of an index’s drug-resistance profile and tuberculosis, without adjusting for the mediating variable of the previous tuberculosis exposure. In this multivariate analysis, we found that the risk of tuberculosis infection was higher among contacts living in the middle of Jiangsu Province (AOR = 7.42; 95% CI = 4.40–12.49; *P* < 0.001) and north of Jiangsu Province (AOR = 3.85; 95% CI = 2.39–6.20; *P* < 0.001 compared with living in the southern region), with > 4 family members (AOR = 1.40; 95% CI = 1.01–1.94; *P* = 0.046 compared with households with ≤ 4 family members), and those exposed to MDR tuberculosis cases (AOR = 1.37; 95% CI = 1.01–1.84; *P* = 0.041). Contacts were at lower risk of tuberculosis infection if they had an independent kitchen (AOR = 0.48; 95% CI = 0.31–0.76; *P* = 0.001).

The direct effect of exposure to a MDR tuberculosis case on contact infection status after adjustment for the mediating variable, previous tuberculosis exposure, radically altered (AOR = 0.55; 95% CI = 0.38–0.81; *P* = 0.003) compared with the indirect effect measured in the univariate analysis (OR = 1.18) and the multivariate analysis not adjusting for previous tuberculosis exposure (AOR = 1.37). In this multivariate model, living in the middle of Jiangsu Province (AOR = 7.97; 95% CI = 4.65–13.68; *P* < 0.001) and north of Jiangsu Province (AOR = 3.87; 95% CI = 2.38–6.32; *P* < 0.001 compared with living in the southern region), > 4 family members (AOR = 1.42; 95% CI = 1.01–1.99; *P* = 0.045 compared with households with ≤ 4 family members), and previous tuberculosis exposure (AOR = 4.87; 95% CI = 3.33–7.13; *P* < 0.001) were remained associated with tuberculosis infection. Contacts remained at lower risk of tuberculosis infection if they lived in a household with an independent kitchen (AOR = 0.55; 95% CI = 0.34–0.88; *P* = 0.012). No statistically significant differences were found between contact BCG vaccination status, the number of bedrooms in the household, and contact age or sex (Table 3).

**Table 3:** Multivariate mediation analyses of risk factors for tuberculosis infection in household contacts of tuberculosis cases

Variable	Multivariable model I: without past tuberculosis exposure included	Multivariable model II: with past tuberculosis exposure included
	Adjusted odds ratio (95% CI), *P* value	Adjusted odds ratio (95% CI), *P* value
Age, years		
< 15	Reference	Reference
15–42	1.17 (0.76–1.79), 0.485	1.14 (0.73–1.77), 0.577
≥ 42	1.21 (0.78–1.89), 0.401	1.13 (0.71–1.80), 0.599
Sex		
Male	Reference	Reference
Female	0.96 (0.71–1.29), 0.773	0.97 (0.71–1.32), 0.847
Region of Jiangsu Province		
South	Reference	Reference
Middle	7.42 (4.40–12.49), < 0.001	7.97 (4.65–13.68), < 0.001
North	3.85 (2.39–6.20), < 0.001	3.87 (2.38–6.32), < 0.001
BCG vaccinated		
Yes	Reference	Reference
No	0.79 (0.55–1.13), 0.198	0.76 (0.52–1.11), 0.151
No. family members		
≤ 4	Reference	Reference
> 4	1.40 (1.01–1.94), 0.046	1.42 (1.01–1.99), 0.045
Independent kitchen		
No	Reference	Reference
Yes	0.48 (0.31–0.76), 0.001	0.55 (0.34–0.88), 0.012
No. bedrooms		
≤ 4	Reference	Reference
> 4	1.01 (0.70–1.45), 0.951	1.06 (0.73–1.55), 0.760
Drug resistance profile of the index case		
Drug-susceptible	Reference	Reference
Multidrug-resistant	1.37 (1.01–1.84), 0.041	0.55 (0.38–0.81), 0.003
Previous tuberculosis exposure		
No	–	Reference
Yes	–	4.87 (3.33–7.13), < 0.001

BCG = Bacille Calmette-Guérin, adjusted for age, sex and drug resistance profile of the index case.

## DISCUSSION

In this large household survey with over 1,000 exposed contacts and almost 500 index cases, we found that contacts of MDR tuberculosis patients were repeatedly exposed to tuberculosis and this strongly mediated the relationship between tuberculosis infection and a source case’s drug-resistance profile. After adjusting for repeated exposures, contacts of DS tuberculosis were at almost two times more likely to have tuberculosis infection. Some studies have shown that exposed contacts of MDR tuberculosis patients are at lower risk for primary progressive disease and our results suggest that they may also be at lower risk for tuberculosis infection after considering recurrent and continuous exposure.

Several previous studies have shown increased rates of tuberculosis infection in contacts of MDR tuberculosis patients compared with contacts of DS tuberculosis patients.^9–13^ In three studies in South Africa, Canada, and Vietnam, contacts of MDR tuberculosis patients had statistically higher levels of tuberculosis infection.^9,12,13^ Two other studies found elevated, but nonsignificant, rates of tuberculosis infection among MDR-TB patients (44% versus 37% in Teixeira and others; ^11^ 17.5% versus 12.1% in Palmero and others^10^), likely because of low sample sizes. Several observations from our study may help to explain these previous findings. First, tuberculosis treatment regimens for MDR tuberculosis patients are rigorous and can be 18 months or longer in some instances. This may have also led unequal exposure periods between groups as shown in our study sample. Second, MDR tuberculosis patients are much more likely to have previous episodes of tuberculosis compared with DS tuberculosis patients. Third, contacts exposed to patients who have developed tuberculosis several times are susceptible to repeated exposure and therefore may eventually succumb to tuberculosis infection or be continuously reinfected. In our study, we found that when we did not adjust for previous tuberculosis exposure, such as in previous studies, tuberculosis infection was greater among contacts of MDR tuberculosis patients. However, on adjusting for mediation between previous tuberculosis exposure and the index’s drug resistance profile, the relative risk of tuberculosis infection in contacts of MDR and DS tuberculosis patients completely reversed and contacts of DS tuberculosis patients were now almost two times more likely to have tuberculosis infection. Prior studies did not adjust for previous tuberculosis exposure and our results suggest the large influence of this mediating factor may have distorted prior study results. Future studies investigating the transmission potential of MDR and DS tuberculosis patients should adjust for prior index tuberculosis episodes.

A recent study conducted by Grandjean and others^8^ in Peru found that the incidence of tuberculosis was lower among 1,055 household contacts of MDR tuberculosis patients compared with 2,362 household contacts of DS tuberculosis patients after three years of follow-up. Tuberculosis infection was not measured (at baseline or during follow-up) in this study and therefore this result may be explained either by an unbalanced underlying prevalence of tuberculosis infection at baseline between groups or a differing susceptibility to primary progressive disease. Our study results suggest baseline tuberculosis infection rates, after accounting for repeated tuberculosis exposure in contacts, may be higher among contacts of DS patients and this may partially explain these findings.^8,12^

We identified other risk factors for tuberculosis infection. Crowding, measured through the number of household family members, has been shown to be an important risk factor for tuberculosis infection in several studies in sub-Saharan Africa.^16,17^ In our study, participants with a large family size had a higher prevalence of tuberculosis infection conflicting with studies coming from low-income areas, such as sub-Saharan Africa.^16,18^ This may be due to the distinct burden of tuberculosis in these settings. Although China has a substantial number of the total tuberculosis cases due to its large population size, the overall incidence of tuberculosis is lower than that of sub-Saharan Africa. In this setting, having more family members may make it more likely to be exposed to the disease. We also found that the presence of an independent kitchen was protective against tuberculosis infection and this variable is likely representative of household socioeconomic status.

There are several limitations to our study. First, although we measured and adjusted for multiple exposures from tuberculosis patients to contacts, the duration of exposure time of household contacts to their respective index patients was not measured and may give a more accurate representation of this mediating factor. Second, tuberculosis infection increases with age as exposures accumulate over time.^17–19^ Nondifferential misclassification may occur if contacts were infected before the current exposure event at earlier ages. A longitudinal tuberculin conversion study may more accurately measure new transmission events and further clarify these issues. Lastly, we used tuberculin skin testing and not interferon-gamma assays to measure tuberculosis infection in this study. A prior study in China found that an agreement between the tuberculin skin test and QuantiFERON-TB Gold In-Tube (QFT, Qiagen, Valencia, CA) was higher in the elderly populations without a BCG scar.^20^ In our study, the proportion of BCG-vaccinated household contacts was similar in both groups and, therefore, any bias due to BCG vaccination is likely nondifferential. Furthermore, we used a 10-mm induration reaction as a positive test to minimize the potential for false-positive tuberculin skin test results.^21^

In conclusion, we found that repeated tuberculosis exposure among contacts of MDR tuberculosis patients strongly mediated the relationship between tuberculosis infection and the index’s drug-resistance profile. Prior studies showing lower rates of incident tuberculosis among MDR tuberculosis patients may be partially explained by elevated rates of tuberculosis infection at baseline.
